# Drug repurposing in amyotrophic lateral sclerosis (ALS)

**DOI:** 10.1080/17460441.2025.2474661

**Published:** 2025-03-03

**Authors:** Emily Carroll, Jakub Scaber, Kilian V. M. Huber, Paul E. Brennan, Alexander G. Thompson, Martin R. Turner, Kevin Talbot

**Affiliations:** a Nuffield Department of Clinical Neurosciences, University of Oxford, Oxford, UK; b Kavli Institute for Nanoscience Discovery, University of Oxford, Oxford, UK; c Centre for Medicines Discovery, Nuffield Department of Medicine, University of Oxford, Oxford, UK; d Target Discovery Institute, Nuffield Department of Medicine, University of Oxford, Oxford, UK

**Keywords:** Amyotrophic lateral sclerosis (ALS), neurodegeneration, drug repurposing, drug discovery, experimental medicine

## Abstract

**Introduction:**

Identifying treatments that can alter the natural history of amyotrophic lateral sclerosis (ALS) is challenging. For years, drug discovery in ALS has relied upon traditional approaches with limited success. Drug repurposing, where clinically approved drugs are reevaluated for other indications, offers an alternative strategy that overcomes some of the challenges associated with de novo drug discovery.

**Areas covered:**

In this review, the authors discuss the challenge of drug discovery in ALS and examine the potential of drug repurposing for the identification of new effective treatments. The authors consider a range of approaches, from screening in experimental models to computational approaches, and outline some general principles for preclinical and clinical research to help bridge the translational gap. Literature was reviewed from original publications, press releases and clinical trials.

**Expert opinion:**

Despite remaining challenges, drug repurposing offers the opportunity to improve therapeutic options for ALS patients. Nevertheless, stringent preclinical research will be necessary to identify the most promising compounds together with innovative experimental medicine studies to bridge the translational gap. The authors further highlight the importance of combining expertise across academia, industry and wider stakeholders, which will be key in the successful delivery of repurposed therapies to the clinic.

## 1. Introduction

Amyotrophic lateral sclerosis (ALS), the most common form of motor neuron disease (MND), is a neurodegenerative disorder for which there is currently no effective treatment. ALS results from the death of motor neurons in the brain and spinal cord, leading to denervation, muscle atrophy and spasticity resulting in progressive paralysis and premature death, typically due to neuromuscular respiratory failure [1]. The median survival time is only 30 months from symptom onset, but there is significant heterogeneity in the rate of progression, with a small proportion (<10%) of individuals surviving for 10 years or more [2], and variation in the relative extent of upper motor neuron (cortical pyramidal cell) and lower motor neuron (ventral horn) involvement. Subgroups are recognized in which loss of function can be restricted for long periods to anatomically restricted regions (arms, legs or bulbar), which are typically slower progressing [3]. The most striking extramotor feature of ALS is the pathological and clinical presence of frontotemporal dementia in a proportion of patients [4].

Treatment to alter the natural history of ALS has proven extremely difficult to develop. Despite over 120 Phase II and III trials in the decade to 2019 [5], only riluzole, developed for ALS nearly three decades ago, is widely licensed for the disease. Notable advances have been made in the development of antisense oligonucleotide (ASO) therapies targeting ALS-associated genes (*SOD1* and *FUS*), but will benefit only a small percentage (<3%) of ALS patients who carry these pathological variants [6]. For the remaining majority of ALS patients with no known underlying genetic cause, there is an urgent need to identify new treatments that meaningfully alter the disease course.

In this review, we discuss the challenge of drug discovery in ALS and explore the potential of drug repurposing in the identification of new effective treatments. We summarize current approaches for drug repurposing, spanning experimental and computational methodologies, and outline some general principles for preclinical and clinical research to help bridge the translational gap.

## 2. Why is drug discovery in ALS so challenging?

A large number of compounds, targeting diverse cellular mechanisms, have been explored for the treatment of ALS. Despite often promising evidence in preclinical disease models, most of these compounds failed to demonstrate clinical benefit when examined in clinical trials [5]. The reasons for these failures are multifactorial and complex, yet ultimately stem from our limited understanding of the causes, initiating factors, and key pathophysiological drivers of ALS [7].

### 2.1. The complex pathway to ALS

A widely accepted model of ALS pathogenesis is based on a ‘multiple-hit’ hypothesis where ALS arises due to the intersection of multiple distinct age-dependent biological events in individuals with an appropriate genetic risk background [8,9]. The stereotyped presentation of the disease could indicate convergence on a set of common cell biological pathways, but also that the neuronal network for voluntary movement has a distinct set of inherent structural and functional vulnerabilities to aging biology. The role of neurodevelopmental factors is of increasing interest but remains poorly defined. Most preclinical research driving the translational pipeline of necessity relies on the use of models based on mutations in the genes linked to ALS, with the potential limitation that the relevance of these models to so-called ‘sporadic’ disease must be considered provisional.

Between 10% and 15% of ALS cases carry a disease-associated genetic variant from a growing list, including *C9orf72*, *SOD1*, *FUS* and *TARDBP* [10–12]. However, the penetrance of disease determining variants in ALS-associated genes can be incomplete, for example ranging from 16% to 60% in the case of the *C9orf72* hexanucleotide repeat expansion [13,14]. Moreover, several ALS-associated variants can lead to different clinical presentations within members of the same family, spanning the clinicopathological spectrum between motor-predominant ALS and frontotemporal dementia (FTD) [15]. Genetic modifiers, including *UNC13A* polymorphisms and *ATXN2* polyQ intermediate repeats, have been shown to increase the risk of developing ALS and modify survival after disease onset [16–22].

The pathological hallmark of ALS is the presence of ubiquitinated, phosphorylated cytoplasmic aggregates of TDP-43 in degenerating neurons, except in a small percentage of patients with *FUS* or *SOD1* variants, whose neuropathology is instead characterized by inclusions containing the variant proteins [23–27]. Specific neuropathological features can be seen in addition to typical TDP-43 pathology with several ALS-causing variants including *C9orf72* and *ANXA11* [28,29]. Widespread disruption to cellular homeostasis involving a range of pathophysiological processes has been identified in motor neurons in ALS. These have been extensively reviewed elsewhere but include glutamate excitotoxicity, mitochondrial dysfunction, oxidative stress, impaired intracellular trafficking and axonal transport, glial cell dysfunction, neuroinflammation, dysregulated nucleocytoplasmic transport, impaired protein homeostasis and ER stress, impaired DNA damage repair, and aberrant RNA metabolism [30]. Given this level of complexity, it is unclear which mechanisms are primary drivers of disease initiation and progression and thus represent the best therapeutic targets. The extent to which different mechanisms operate between patients is also unclear and may ultimately require an individualized approach to therapy.

### 2.2. Challenges in modelling the disease

The distinctly human nature of ALS is hypothesized to arise from factors which include the rapid increase in brain volume and connectivity in recent hominin evolution, including lateralization of cerebral functions, and human specific genomic variation such as genetic founder effects in the human population [31–33]. TDP-43 nuclear depletion results in mis-splicing and de-repression of cryptic spice sites, in patterns which will be species specific [34]. Findings of disease-relevant human variation in *UNC13A*, one of the human specific targets of TDP-43, have given further support for the importance of this mechanism in the development of ALS [35].

As animals do not appear to develop ALS spontaneously, with the possible exception of SOD1 variants causing ALS-like degenerative myelopathy in dogs [36], *in vivo* models of ALS rely on the introduction of human ALS-associated genes into the host genome through random or targeted, site-specific, integration. The *SOD1*^*G93A*^ mouse model [37] has been extensively used for drug testing over three decades and was pivotal in providing *in vivo* evidence for *SOD1* antisense oligonucleotides [38]. However, compounds demonstrating a neuroprotective effect in this mouse model have generally failed to translate to a clinical benefit in humans [39]. Forcing a phenotype through gene overexpression raises questions about the physiological relevance to human ALS [40]. Subsequently, as other genes have been identified, multiple models carrying human *TARDBP*, *FUS* or *C9ORF72* ALS-causing variants have been created with different methods and variable phenotypes [41–45]. Additionally, ‘humanised’ mouse models have been developed, using targeted replacement of mouse genomic regions with orthologous human sequences [46], which in principle preserve physiological levels and expression of the gene of interest. However, it is not clear that these models consistently develop a degenerative disease of the motor system, potentially limiting their usefulness [47].

In contrast to *in vivo* models, *in vitro* models provide the capacity to investigate the cellular and molecular changes underlying ALS at a larger scale, with applications to high-throughput drug screening. Transformed cell-line models (including HEK cells, HeLa cells and motor neuron-like NSC34 cells) and motor neuron models such as mouse primary motor neuron cultures, organotypic cultures, and mouse stem cell-derived motor neuron models have been used extensively [44,48,49]. The relevance of these model systems to the human disease is necessarily indirect. Advances in stem cell technology have led to the development of human induced pluripotent stem cell-derived motor neuron (iPSC-MN) models of ALS [50,51]. Although currently the only approach available to model sporadic ALS, successful translation of findings from iPSC-MN models to treatments for ALS patients are still lacking. The relative immaturity of iPSC-derived neuronal cultures is highlighted by comparative transcriptomic analysis, showing a closer resemblance to fetal than adult spinal tissue [52,53]. A high level of technical and biological variance from factors not directly related to ALS mechanisms exists between different lines and across experiments using the same lines, leading to significant problems in reproducibility [53–55]. Co-culture models incorporate additional supporting cells in the CNS such as microglia and astrocytes and allow the possibility of using microfluidics for compartmentalization to allow modeling of neuromuscular junctions or other specific cellular interactions [56,57]. Additionally, organoids have been developed from human iPSC-MNs, which incorporate several different cell types including motor neurons, glia, sensory neurons as well as skeletal muscle and vasculature [58,59].

As no single model has been shown to demonstrate clear therapeutic predictive value, testing drug efficacy across a series of models, each reflecting a different aspect of the disease pathophysiology, is likely to be the best approach to prioritizing the most promising candidates for further development.

## 3. The potential of drug repurposing in ALS

### 3.1. Drug repurposing versus novel drug discovery

Developing a new drug from initial concept through to the launch of a new product is a time-consuming and expensive process, which can take over 10 years and cost billions of pounds. Such *de novo* drug discovery approaches are also associated with a high risk of failure. The success rate for CNS drugs is substantially lower and the drug discovery process longer, than that of non-CNS drugs [60].

Drug repurposing involves evaluating the ability of existing drugs to treat additional disorders distinct from their original indication. This approach stems from the fact that many biological targets have multiple roles in disparate pathologies [61], and observations that many drugs act on multiple targets, a characteristic referred to as polypharmacology. These ‘off-target’ effects arising from nonselective binding often underlie side effects but, in some cases, may also generate additional therapeutic potential [62,63]. Polypharmacological properties of drugs may also be particularly beneficial in complex disorders such as ALS, where simultaneously targeting multiple pathophysiological processes contributing to neurodegeneration may be desirable. Furthermore, given the lack of clarity around a unifying ‘cause’ of ALS and few specific therapeutic targets, repurposing approaches provide an effective approach to screen large numbers of clinically approved compounds for neuroprotective properties. Drug repurposing also offers significant advantages over traditional drug discovery approaches. Repurposed drugs are associated with shorter timeframes and a reduced risk of failure as preclinical testing and compound safety have already been ascertained, resulting in a reduced drug development cost of currently approximately $300 million, compared with an estimated $2–3 billion for *de novo* drug discovery approaches [64]. Therefore, although it also comes with significant challenges, drug repurposing should not be considered a last resort for drug discovery in ALS, but a complementary approach, which provides an opportunity to reexamine the potential of a wide variety of drugs that have already been tested for basic safety.

### 3.2. Current approaches to drug repurposing in ALS

**Figure 1.**
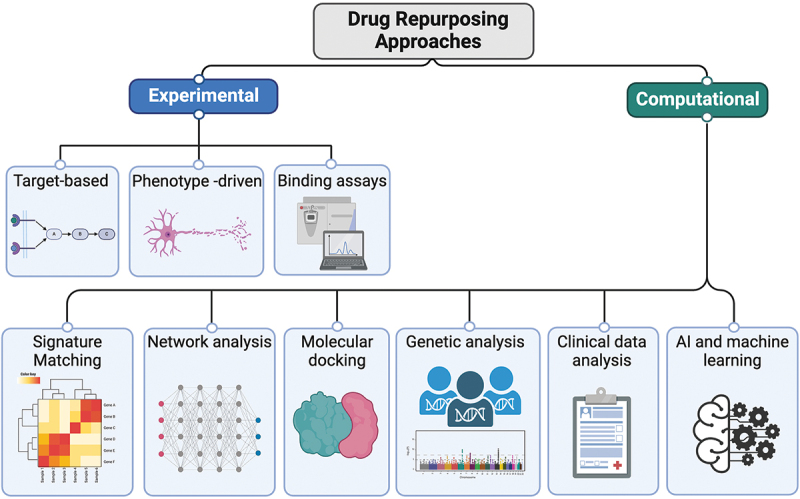
Drug repurposing opportunities can be evaluated using several experimental and computational approaches. Experimental approaches include target-based assays, phenotype-driven assays and binding assays. Computational approaches include signature matching, network analysis, molecular docking, genetic analysis, clinical data analysis and AI and machine learning approaches.

Historically, drug repurposing often occurred when new indications for drugs were discovered by chance. Aspirin is the exemplar: an analgesic now far more commonly repurposed as an anti-platelet agent [65]. Minoxidil, initially developed for hypertension, is now used in the treatment of hair loss [66]. Sildenafil, initially indicated for angina was discovered by trial participants to improve erectile function [67], and thalidomide, initially developed for the treatment of gestational morning sickness (and later withdrawn on discovery of its potent teratogenicity), is now indicated for the treatment of multiple myeloma and erythema nodosum leprosum (ENL) [68]. In the case of neurodegenerative disorders, the anti-viral drug, amantadine, was repurposed for the treatment of Parkinson’s disease following observations of improved symptoms in patients taking the drug for the treatment of flu [69]. Amantadine is now being investigated for repurposing in ALS as part of the MND-SMART trial (NCT04302870). Such serendipitous discoveries have encouraged more intentional drug repurposing strategies to identify ‘new’ uses for ‘old’ drugs (Figure 1). These approaches can be broadly divided into experimental approaches or computational approaches.

#### 3.2.1. Experimental approaches

Experimental approaches for drug repurposing in ALS are reliant on disease models that recapitulate ALS pathophysiology, which in the early stages of drug discovery are usually cellular and can be rapidly screened with large, repurposed drug libraries. Drug screening in cell models can be performed by target-based approaches, phenotype-driven approaches and binding assays.

##### 3.2.1.1. Target-based approaches

Target-based approaches involve identifying a key molecular substrate or pathway implicated in ALS pathophysiology and directly testing candidates known to engage this target, or screening compounds predicted to modulate the target.

To date, many of the drugs currently under clinical development for repurposing in ALS have been identified using a target-based approach (Table 1). One example is the alpha-1 adrenergic receptor antagonist, terazosin, which is currently under investigation for ALS in an experimental medicine study. Terazosin is an FDA approved drug, prescribed for the treatment of benign prostatic hyperplasia and hypertension. However, through off-target effects terazosin increases the activity of the key glycolytic enzyme, phosphoglycerate kinase 1 (PGK1). Substantial literature suggests that bioenergetic dysfunction is a key pathophysiological process underlying neurodegeneration in ALS [70]. Moreover, downregulation of PGK1 has previously been reported in astrocytes from the SOD1^G93A^ mouse model [71] and fibroblasts from ALS patients [72]. It was hypothesized that increasing the activity of PGK1 using terazosin may be therapeutically beneficial. Indeed, terazosin was found to be neuroprotective in ALS models [73], and in models of stroke [74] and in spinal muscular atrophy [75]. Terazosin has also shown therapeutic effects in Parkinson’s disease models and in patients with Parkinson’s disease [76]. Patients prescribed terazosin, or other drugs that exert similar effects on the activity of PGK1, visited hospital less often and demonstrated improved motor and non-motor symptoms [76]. Based on this preclinical evidence, an experimental medicine study was designed to examine the effects of terazosin in 50 ALS patients (Terazosin RepUrposing STudy in ALS (TRUST) trial), with results expected in 2025 (ISRCTN45028842).

**Table 1.** Overview of current drugs under clinical investigation for repurposing in ALS.

Drug	Initial Indication	Pathogenic mechanism targeted in ALS	Stage of development	Reference
Amantadine	Antiviral	Excitotoxicity	Phase II/III	NCT04302870
Ambroxol	Mucolytic for the treatment of respiratory disorders	Oxidative stress, dysregulation of sphingolipid metabolism	Phase II	NCT05959850
Baricitinib	Rheumatoid arthritis, Eczema (Europe and Japan)	Inflammation	Phase I/II	NCT05189106
Bosutinib	CML	Proteostasis	Phase I/II	NCT04744532
Ciprofloxacin and Celecoxib	Antibiotic (ciprofloxacin)and NSAID (celecoxib)	Inflammation	Phase II	NCT05357950
Darifenacin	Incontinence	NMJ dysfunction	Phase II	NCT06249867
Dolutegravir, abacavir and lamivudine (Triumeq)	HIV	HERVK expression	Phase III	NCT05193994
Fasudil	Subarachnoid haemorrhage	Inflammation	Phase II	NCT05218668
Ibudilast	COPD	Neuroinflammation and microglial activation	Phase IIb/III	NCT04057898
IL-2 and Abatacept	IL-2 (Metastatic melanoma, metastatic renal cell carcinoma) Abatacept (rheumatoid arthritis)	Inflammation	Phase I	NCT06307301
Mastinib	Mastocytosis, severe asthma	Neuroinflammation (microglia)	Phase III	NCT03127267
Metformin	Diabetes	Proteostasis	Phase II	NCT04220021
Monepantel*	Anthelmintic	Proteostasis	Phase I	NCT06177431
Lithium carbonate	Depression, bipolar disorder	Synaptogenesis, autophagy	Phase III	NCT06008249
Terazosin	Hypertension and benign prostatic hyperplasia	Metabolic dysfunction	Experimental medicine study	ISRCTN45028842

Data from ClincalTrials.gov with trials classed as ‘active, not recruiting’ or ‘recruiting’ and the ISRCTN registry in January 2025. COPD: chronic obstructive pulmonary disease; CML: chronic myeloid leukemia; NSAID: non-steroidal anti-inflammatory; NMJ: neuromuscular junction; HIV: human immunodeficiency virus. HERVK: human endogenous retrovirus K. * veterinary drug.

However, drug repurposing via target-based approaches has yet to result in the development of a successful therapy. Edaravone, a free radical scavenger used for the treatment of stroke that was hypothesized to target oxidative stress in ALS. A Phase III double-blind placebo-controlled study found no significant effect of edaravone on ALSFRS-R scores [77], however post-hoc analysis and a subsequent double-blind placebo-controlled Phase III trial indicated that edaravone may have beneficial effects in a subset of ALS patients with greater baseline functionality [78]. On this basis, edaravone was licensed for use in Japan in 2015 and in the United States in 2015 [79]. However, the European Medicines Agency declined to license edaravone in 2019, citing insufficient evidence of long-term efficacy (EMA review document (CHMP/290284/2019)). In 2024, it was reported that administration of the oral edaravone formulation, FAB122, failed to meet its primary or secondary endpoints in a Phase III clinical trial (ADOREXT: NCT05866926) [80].

There are several other similar examples where despite promising initial early evidence, drugs were ultimately found to have no significant effect on slowing disease progression in clinical trials including: arimoclomol, a heat-shock protein (HSP) co-inducer thought to assist in protein clearance [81,82]; memantine, an anti-glutamatergic agent approved for the treatment of Alzheimer’s disease that reduces glutamate excitotoxicity [83], the antibiotic minocycline that has been shown to reduce inflammation and apoptosis *in vitro* [84], and the combined treatment of sodium phenylbutyrate and taurursodiol to target ER stress and mitochondrial dysfunction [85,86] (Table 2).

**Table 2.** Examples of repurposed drugs that failed to demonstrate efficacy in clinical trials.

Drug	Initial Indication	Pathogenic mechanism targeted in ALS	Stage of development	Reference
Arimoclomol	Insulin resistance, complications of diabetes mellitus	Impaired proteostasis	Phase III	[1]
Dimethyl fumarate	Multiple sclerosis	Inflammation	Phase II	[2]
Edaravone	Stroke	Oxidative stress	Phase III	[3,4]
Memantine	AD	Glutamate excitotoxicity	Phase III	[5]
Minocycline	Antibiotic	Inflammation	Phase III	[6]
Perampanel	Partial onset seizures	Glutamate excitotoxicity	Phase II	[7]
Rasagiline	PD	Oxidative stress and apoptosis	Phase II	[8]
SB-TURSO	SB: urea cycle disorders TURSO: gallstones	ER stress and mitochondrial dysfunction	Phase III	[9]
Trazodone	Depression	Modulation of the UPR	Phase III	[10]

Data from PubMed searches, ALZforum and the MND Association. AD: Alzheimer’s disease; PD: Parkinson’s disease; SB: sodium phenylbutyrate; TURSO: taurursodiol; ER: endoplasmic reticulum; UPR: unfolded protein response.

These findings underpin the complexity of the disease, as all the aforementioned examples target different biological pathways implicated in ALS pathogenesis. A major limitation of drug repurposing through target-based approaches is that this relies upon existing knowledge and may miss opportunities to identify new therapeutic targets. A greater understanding of the sequence of events culminating in motor neuron loss is required before target-based approaches to ALS drug discovery can achieve their full potential. With incremental advances in our understanding of ALS disease mechanisms, new molecular targets emerge continuously, exemplified by current studies looking to modify the aberrant splicing resulting from TDP-43 depletion [35,87,88]. As new targets are identified, online databases such as ‘The Drug Repurposing Hub’ developed by ‘The Broad Institute’ will be immensely valuable to identify existing drugs that are known to target certain genes and cellular pathways [89].

##### 3.2.1.2. Phenotype-driven approaches

Phenotype-driven drug screening is a target-agnostic approach, where understanding the underlying disease pathophysiology is not essential. This approach can be defined as a screening method to identify compounds which induce detectable changes in a biological model of disease. This is an attractive approach in ALS, given the lack of a clarity about tractable therapeutic targets. Phenotypic readouts can range from molecular and cellular phenotypes in *in vitro* models, through to behavioral readouts in *in vivo* models such as mice and zebrafish.

Phenotypic drug screens can also uncover previously unknown molecular mechanisms, signaling pathways or therapeutic targets. For example, a phenotypic drug screen in iPSC-derived motor neurons expressing a *SOD1* mutation has previously been performed to examine the effects of 1,416 compounds already approved for the treatment of other diseases or undergoing clinical testing. From the initial screen, 27 primary hits were identified to increase survival relative to negative controls. Of these 27 primary hits, 14 were found to converge on inhibiting the Src/c-Abl signaling pathway, including bosutinib, a drug approved for the treatment of chronic myelogenous leukemia. In subsequent assays, bosutinib was found to increase autophagy, reduce the amount of misfolded mutant SOD1 protein and attenuate aberrant expression of mitochondrial genes [90]. These promising preclinical findings led to a small scale, placebo-controlled Phase I clinical trial of bosutinib in which it was found to be safe and well tolerated, with a subset of patients with lower levels of plasma neurofilament light chain (NfL) appearing to show slowed rates of progression. However, larger clinical trials which evaluate efficacy are awaited to determine whether bosutinib warrants further development for ALS [91].

Such studies highlight the value of phenotype-driven approaches in the ability to identify previously unknown drug targets and mechanisms of action that would be missed by target-based approaches. However, phenotype-driven approaches are heavily reliant on using physiologically relevant model systems that reflect the human disease to ensure findings in preclinical models can be translated to clinical benefit.

##### 3.2.1.3. Binding assays to identify drug targets

A comprehensive analysis of drug-binding sites is lacking, suggesting there may be missed opportunities for drug repurposing. Several chemical proteomic techniques have been developed to allow drug target identification, which involve affinity chromatography coupled with mass spectrometry and bioinformatic analyses. Chemical proteomic techniques for target identification can be broadly divided into activity-based protein profiling (ABPP) and compound-centric chemical proteomics (CCCP). ABPP involves the use of an affinity probe which includes a reactive warhead together with a reporter element, typically a fluorescent group, biotin, alkynes or azide, to visualize protein targets. The probe can then be incubated with cells, lysates or tissue homogenates *in vitro*, in absence or presence of the drug of interest, and after enrichment with chemical or biochemical techniques, the protein targets of the drug can be identified with proteomic approaches such as mass spectrometry. CCCP methods are similar in principle, except the drug of interest is modified to incorporate an affinity handle to allow immobilization of the drug on a matrix support, such as magnetic or agarose beads. Subsequently, cell lysates or tissue are incubated with the matrix and washed to remove nonspecific binders. After complete elution, the enriched proteins can be identified with proteomics techniques as with ABPP [92]. Alternatively, so-called label-free approaches such as the Cellular Thermal Shift Assay (CETSA) maybe employed. CETSA is based on the biophysical principle of ligand-induced thermal stabilization of target proteins. In this assay, cells are treated with the drug of interest, then heated to denature and precipitate proteins. The principle of this assay is that unbound proteins will denature and precipitate when heated, whereas bound proteins remain in solution. The identity of bound proteins can then be established with proteomics [93,94].

Binding assays have the potential to be immensely valuable for drug repurposing. Whilst compounds identified in a repurposed drug screen will have a proposed target and mechanism of action linked to their original indication, whether these compounds are exerting protective effects via this mechanism or through a non-canonical pathway is important to establish. However, the techniques are challenging in practice, require extensive downstream validation, and conjugation of a drug with a detection moiety can alter its function and binding specificity. Additionally, coverage of membrane protein targets using proteomic approaches remains a challenge.

#### 3.2.2. Computational approaches

Computational approaches integrate data from various sources to help formulate research hypotheses and identify drugs with potential for repurposing. In some cases, these data are derived from cellular or animal models of the disease whereas other approaches involve the analysis of clinical datasets. Common computational approaches and their feasibility for use in drug repurposing for ALS are discussed below.

##### 3.2.2.1. Signature matching

Signature matching uses representations of disease states, including transcriptomic (RNA), proteomic or metabolomic data from disease models, and seeks to identify drugs capable of reversing disease-associated signatures. The transcriptomic landscape of ALS has been extensively analyzed using cell models and postmortem tissue across a range ALS-associated mutations and in sporadic ALS [95–101]. Large-scale bulk RNA sequencing approaches have been used to examine expression profiles across hundreds of controls and patient-derived iPSC-MNs and in spinal cord tissue. These studies have identified certain disease-associated ‘signatures,’ including activated DNA damage responses, P53 activation and upregulation of inflammatory pathways linked to TNF and NF-κβ [53,102].

Transcriptomic approaches can also be used to develop drug-associated signatures, for example from a cell line following systematic treatment with different pharmacological compounds. ‘Connectivity Map’ or CMap, and the scaled-up version termed the Library of Integrated Network-based Cellular Signatures (LINCS) database, provide a comprehensive library detailing the effects of genetic and pharmacologic perturbations across multiple cell types on transcriptomic cellular signatures [103,104]. This vast database provides an invaluable resource for drug repurposing, as drug-associated signatures that appear to oppose disease-associated signatures might indicate compounds with potential for repurposing in that disease. For example, in Alzheimer’s disease (AD), the CMap database has been used to identify small-molecule compounds for drug repurposing using transcriptomic data generated from postmortem AD patient brains and murine AD brain samples, to select a set of 153 drugs predicted to reverse or oppose disease-associated changes. Downstream analysis of these compounds in iPSC-derived cortical neuron cultures found that 51 drugs from the original 153 identified demonstrated opposite effects to that of the AD associated transcriptomic signature [105]. Furthermore, the NeuroLINCS Centre has been established with a specific focus on the development of a signature database for ALS, where they aim to combine iPSC technology, transcriptomics, epigenomics, metabolomics and proteomics to generate disease signatures across different cell types [106].

Transcriptomic signatures of disease states have significant limitations. Transcriptomic data from model organisms is limited by effectiveness in modeling the disease, which has already been discussed in detail above. Disease signatures from *post-mortem* tissue also have significant limitations, as they often no longer contain the cell of interest (which has degenerated) and may have a significant contribution from secondary effects [107]. A possible way to overcome this is to use genome-wide association studies (GWAS) to create a model of genetically regulated expression, without measuring the effect of the GWAS SNPs using RNASeq. This method, known as transcriptome-wide association study (TWAS) relies on the premise that genetic variants explain most global transcript variation. This model can be used for signature matching, much like disease state expression changes can, and has been successfully used in ALS [108]. Limitations of this approach include the usual concerns about linkage of SNPs in GWAS studies, exclusion of methylation and environmental factors from this model, as well as over-reliance on cis-acting expression quantitative trait loci (qQTL) [109].

The assumption underlying all signature-based approaches is that the reversal of these signatures predicts clinical benefit. However, changes in expression that are observed in cell models of disease may not be directly correlated with the changes in expression observed in human disease tissue and may also be compensatory not harmful. Furthermore, how disease signatures in cell models relate to behavioral or anatomical phenotypes observed in mouse models or clinical observations in human patients are unclear. Given this, investigating transcriptomic changes in the brains of ALS mouse models, both with and without drug treatment, and matching these signatures with behavioral and anatomical readouts is important to enhance the potential of signature matching as a tool for drug repurposing. Single cell-based methods to study drug-induced changes on specific cellular populations of interest will likely also provide further granularity to inform drug repurposing [110].

##### 3.2.2.2. Network analysis

Network medicine is an emerging field based on the premise that a disease phenotype rarely stems from the dysfunction of a single gene or protein but instead results from the dysfunction of a wider complex network of genes or proteins intricately interconnected to each other. This network encompasses both intracellular and intercellular connectivity, meaning that the effects of a single genetic mutation are not necessarily restricted to the cell that carries it, but may affect gene expression more widely across other cells in the network. In the context of disease, genes that are linked to a particular disease tend to cluster within a particular region of the network and form ‘disease modules’ [111]. Similarly, introducing drugs into the network leads to alterations that can be mapped and their proximity to disease modules studied. The proximity of a drug target to a disease module can be used to predict the effectiveness of a drug in the treatment of that disease [112–114].

Network-based algorithms have been used to identify potential off-label use of drugs in ALS. A network-based algorithm called SAveRUNNER can interrogate existing datasets detailing the human protein–protein interactome, disease-associated genes and drug–target interactions. SAveRUNNER applies proximity analysis, to quantify the distance between ‘disease modules’ and ‘drug modules’ to investigate associations between ALS-associated genes and drug targets identified within the human protein interactome. This led to the identification of over 400 drugs with potential for drug repurposing in ALS including histaminergic compounds, cyclooxygenase enzymes, and benzodiazepines [115].

Network-based approaches can also be combined with signature mapping approaches. This has been used to generate a network called MANTRA, whereby compounds with similar expression profiles in CMap were grouped into clusters of drugs with shared modes of action. MANTRA was used to identify a new mechanism of action for the Rho-kinase inhibitor, Fasudil, which showed similarity to the autophagy inducer, 2-deoxy-D-glucose (2-DG). The ability of Fasudil to induce autophagy was confirmed in human fibroblasts and the authors concluded that it could be repositioned as an autophagy enhancer in the treatment of several neurodegenerative diseases [116]. Fasudil is currently still under development as a potential therapeutic for ALS, and in preliminary data from a Phase II trial, treatment was reported to slow motor neuron loss when compared with placebo treated controls [117].

Network-based algorithms could also help stratify treatments across different patient subgroups, depending on the underlying genetic susceptibilities and unique vulnerabilities within the network. As these approaches infer effectiveness through proximity, verification of the outputs from such network-driven methodologies using other modalities is essential to confirm compounds which are identified are indeed capable of modifying ‘disease modules.’ Network biology frequently relies on externally derived datasets, including protein interaction experiments often performed in orthologous species, which may not apply to the disease model in question. There has been a rapid proliferation in network selection methods and external datasets with different strengths and weaknesses, varying performance and discordant results [118]. Expert selection of the correct methodology and appropriate datasets is therefore key to enable detection of disease relevant signatures.

##### 3.2.2.3. Molecular docking

Molecular docking is a structure-based computational approach used to interrogate binding site complementarity between a drug and a target site. Conventional docking, which involves investigating multiple ligands against a single target, can be used when the underlying target is known (for example a disease-relevant receptor or protein). Alternatively, inverse docking approaches can be used to examine multiple target sites across a range of drugs within a large drug library. The latter approach has been used to develop a novel proteo-chemometric method to investigate drug–target interactions and predict new uses for existing drugs. In this study, 3,671 FDA approved compounds were screened across more than 2,000 human protein crystal structures and predicted several drug-target associations that may suggest new disease indications for these compounds. Furthermore, this approach provided insight into the mechanism of action of certain compounds. For example, the anti-parasitic compound, mebendazole, which also demonstrates unexpected anti-cancer properties, shows the structural potential to inhibit VEGFR2, a mediator of angiogenesis which plays a critical role in cancer growth. The binding potential between mebendazole and VEGRF2 was confirmed experimentally [119]. This method may be extended to almost the entire human genome by using predicted structures generated with AlphaFold 2 [120].

The use of *in silico* docking approaches has been used to successfully identify new small-molecule compounds that bind within the RNA recognition motif and N-terminal domains of the RNA-binding protein, TDP-43, which has been heavily implicated in ALS pathogenesis [121,122]. However, the use of such approaches to identify existing drugs that may bind to TDP-43, and therefore have potential in drug repurposing, has not yet been explored. The value of molecular docking approaches in drug discovery for ALS is also somewhat limited by the lack of a specific, and safe, underlying target for therapeutic intervention. TDP-43 is developmentally regulated protein that is essential for early embryonic development and has multifaceted roles in regulation RNA metabolism, suggesting caution must be taken when attempting to target this protein therapeutically [123,124]. However, such approaches may become more valuable as a tool for drug repurposing as the mechanisms underlying the disease become more clear and therapeutic targets emerge. Additionally, if compounds are found to be effective using other approaches, such as experimental phenotypic screening, molecular docking could be used to interrogate the mechanism of action underlying the observed effects. While there has been significant technical advance, particularly with the integration of advanced computer models with artificial intelligence methodologies, the accurate prediction of the dynamic behavior of proteins and the identification of unresolved folds remains a substantial challenge.

##### 3.2.2.4. Clinical data analysis for drug repurposing

Retrospective analysis of clinical data can provide opportunities to identify new indications for existing drugs. Clinical data can be obtained from various sources including electronic health records (EHRs) and self-reported data.

EHRs contain a wealth of data on patient outcomes over the disease course. These data include diagnostic and pathophysiological data (the results of laboratory tests), drug prescriptions (with start and end dates), clinical descriptions of patient symptoms and imaging data. This vast database of both qualitative and descriptive data provides a valuable source of information that can be utilized for drug repurposing [125]. Large-scale pharmacoepidemiological studies using EHRs including prescription data have yielded potential candidate treatments in other disease areas, including other neurodegenerative diseases. Overcoming issues of bias and confounding inherent to observational studies remains a major problem when interpreting such data: early surveillance studies suggesting that statins increased risk of ALS overlooked the association between low-density lipoprotein cholesterol that likely drove the observed association [126,127].

One approach to circumvent these issues, termed clinical genomics signature-based prediction for drug repurposing (ClinDR), combined EHR data with genomic data from public resources to identify similarities between pairs of drugs and diseases and thereby predict novel indications for licensed drugs. Using this approach, they predicted the β-2 adrenoceptor agonist, terbutaline sulfate (TS), widely used for the treatment of asthma, could be repurposed for ALS. The potential of TS was subsequently validated in an *in vivo* zebrafish model of ALS, where treatment with TS was found to prevent neurodegeneration-like phenotypes in this model in a dose-dependent manner [128].

Self-reported patient data can also provide a source of information for drug repurposing. With serious diseases, such as ALS, patients may experiment with drugs that have not yet received regulatory approval. Online patient communities can provide data to monitor the effects of drug use on factors such as symptoms and disease progression. In a previous study data reported on the website PatientsLikeMe by patients with ALS was used to evaluate the effects of lithium carbonate. Self-reported data included ALSFRS scores, symptoms, treatments (with start and stop dates), site of ALS onset and demographic data. Of the 4,318 patients registered on the website, 348 (9%) reported taking lithium, but after inclusion and exclusion criteria were applied, 149 patients remained for subsequent analysis. An algorithm was applied to generate ALS controls not taking lithium matched for each patient taking lithium, based on similar ALSFRS trajectory, and at 12 months identified no effects of lithium treatment on disease progression [129]. However, lithium treatment has subsequently been shown to have potentially neuroprotective effects in a subset of ALS patients and is currently being explored in a Phase III clinical trial (NCT06008249). This highlights the challenges of using self-reported data, which is limited in its value due to inherent issues with data collection. Even though the authors developed an algorithm to generate appropriate controls, the lack of blinding to treatment group, lack of randomization to treatment and missing data due to omissions or inconsistencies in data reported by patients mean that drawing any strong conclusions on this data alone is challenging. However, self-reported patient data may provide a valuable adjunct tool to accelerate clinical discovery and evaluate the effects of drugs known to be safe and already in clinical use.

Clinical datasets can be a valuable resource to inform drug repurposing in ALS, which has certain advantages over modeling approaches. It is estimated that only approximately 30% of compounds that show therapeutic potential in cell models work well in animal models, and that of these, only 5% translate into efficacy in human subjects [130]. Given this, data collected directly from patients is an important source of information, which may help bridge the translational gap in the development of new treatments for ALS.

##### 3.2.2.5. Genetic data for drug repurposing

Data from genetic studies, particularly GWAS studies, can be used directly to find drugs that may target the mutation or its eQTL, or by utilizing the strength of transcriptome-wide association studies as previously discussed. Genetic epidemiological approaches achieve their full effect in combination, such as Mendelian randomization (MR), a powerful method to study causal effects of a broad range of exposures – including environmental or other modifiable factors, levels of proteins or metabolites – on risk or progression of disease [131]. Using the random assortment of alleles during reproduction, MR circumvents confounding effects that dog observational studies. Commonly employed MR methods based on summary data from genome-wide association studies (GWAS; summary MR and two sample MR) also benefit from the large sample sizes typically used in GWAS to study causal effects in rare diseases.

MR using cis-eQTL of druggable gene and ALS GWAS data collections has been used to identify therapeutic gene targets and subsequently candidates for drug repurposing [132]. Through MR analysis, several ALS druggable genes in the brain and blood were identified, including *TBK1, TNFSF12, RESP18, and GPX3*, which were confirmed as targets following further colocalization analysis. Through searching ChEMBL, DrugBank Online and previous publications, five drugs acting on these gene targets were selected. One of these drugs was the FDA-approved TBK1 inhibitor, fostamatinib (AMX), which is indicated for the treatment of immune thrombocytopenic purpura. The neuroprotective effects of fostamatinib were validated using *in vitro* pharmacological assays, where the drug was found to inhibit the cGAS/STING signaling pathway. Additionally, another FDA-approved compound, R788, which acts as a SYK inhibitor and shows high affinity with TBK1, was also shown to inhibit cCAS/STING activation. This pathway has been implicated in neuroinflammation in SOD1 and TDP-43-associated ALS pathogenesis resulting from mitochondrial DNA release [133]. These findings demonstrate how MR techniques can be used to alongside *in vitro* validation to identify new therapeutic targets and drug repurposing opportunities. Drug target approaches combined with phenome-wide association study – in which drug target *cis* eQTLs are tested for associations with a wider range of diseases and other phenotypes – have also been used to identify novel indications for drugs or potential adverse effects [134].

MR methods using the genetic determinants of RNA, protein or metabolite abundance (i.e. eQTLs) as instrumental variables can be employed to provide causal evidence for potential targets identified in models, or to identify novel drug targets. Drug target MR uses as instrumental variables *cis* eQTLs of the protein drug target (i.e. eQTLs within or in close proximity to the gene encoding the drug target) to examine the effect of levels of the drug target as a proxy for drug effect [134]. In the case of ALS, drug target MR has been used to indicate a potential protective effect for apolipoprotein B lowering drugs on risk of ALS [135,136].

GWAS analysis in ALS has largely focussed on identifying the mediators of ALS risk rather than measures of disease aggressiveness (such as survival or rate of disability progression). The limited available evidence suggests that aggressiveness and risk may have different genetic determinants [137] and that the heritability of survival in ALS may be negligible, limiting the power of this approach [138]. It is also important to recognize that the genetic variants utilized in MR are usually common variants which only explain a small proportion of ALS risk, and which typically exert small effects throughout life, potentially during critical periods of development that would not necessarily be replicated by treatment during the symptomatic period [134]. Drug target MR is limited through its exploration of drug *target* (pQTL) effects rather than direct drug effects, meaning that off-target effects, whether adverse or beneficial, are not measured [139]. Additional limitations of drug target MR are the restricted genetic ancestral background of most GWAS, potentially limiting the generalizability of findings, and the underlying assumptions and analytical quality of exposure and outcome GWAS [140].

Overall MR has potential roles in target validation and off-target effects (both novel indications and adverse effects). At present, its usefulness in target identification is primarily directed toward influencing ALS risk; larger studies of the genetic drivers of progression may reorient this to the symptomatic period.

##### 3.2.2.6. AI approaches

Advances in machine learning algorithms, neural networks and large language models (LLMs) have opened entirely new opportunities for drug discovery, with claims that the technology could reduce drug development costs by 25% [141].

The versatility of AI models underlies their applicability to multiple stages in the drug development process including target identification and prediction of druggability, high-throughput screening, structure-based drug design including *de novo* drug design as well as identification of subpopulations who may benefit from certain drugs [142]. Multiple AI-designed compounds for conditions ranging from neoplastic disorders to autoimmune disorders have entered various early stages (Phase I/II) of clinical trials [143]. There are also examples of how such approaches have been used to identify potential ALS therapeutics, including the PIKfyve inhibitor, VRG50635, discovered through Verge Genomics’ AI pipeline, which has completed early safety studies, and is currently recruiting into Phase Ib [144].

While discussion of *de novo* drug design using AI is beyond the scope of this article, AI methodology has also been used specifically in drug repurposing pipelines, with an early example being the discovery of the antimicrobial properties of halicin, originally developed as an antidiabetic drug [145]. The ability to combine data-driven multi-omics approaches with language models that enable sifting through large amounts of existing publication data is a particular strength of AI over existing computational models. An example of such an approach for ALS is the proprietary cloud-based platform PandaOmics (Insilico Medicine), which was based on the dataset generated by Answer ALS [146], which combined deep phenotypic data from the clinic with extensive multiomic data generated from patient-derived iPSC lines. PandaOmics confirmed 17 gene targets already identified by other means, but also discovered 11 novel therapeutic targets, some of which already have known pharmacological compounds that can modulate their action [147]. An open science approach that used the same Answer ALS dataset but instead used the NetraAI platform was able to confirm many of these targets while adding others and also offered *in silico* prediction for patient subgroups most likely to respond to certain target modulations [148].

As the healthcare industry continues to produce large amounts of data [149], AI tools that can integrate and understand large volumes of data will become an increasing part of the drug discovery process, but does, however, rely on the accuracy and relevance of the data. Moreover, the lack of transparency of the method itself, as well as the commercially sensitive nature of much of the data, risks limiting the openness of the platforms, generating further difficulties in the assessment of the validity and utility of AI [150].

## 4. Bridging the translation gap in drug repurposing for ALS

### 4.1. Synergistic approaches using multiple model systems

The biggest challenge in developing repurposing strategies in ALS is the successful translation of promising findings from preclinical models across to meaningful clinical benefit in ALS patients. To date, there are no *in vitro* or *in vivo* models which have proven to be predictive of efficacy in ALS. It is likely that integration between the available range of experimental and computational methodologies, each encompassing its own advantages and limitations, will be synergistic and therefore more powerful. Widely available resources such as CMap combined with pharmacogenomic approaches and validation of findings using computational approaches in preclinical models is essential prior to clinical evaluation.

The selection of models and phenotypes for screening and validation of potential compounds is critical. This will be a balance of tractability versus disease relevance. To be a valuable readout for drug screening a phenotype must be consistent and robust. However, many of the more physiologically relevant preclinical models, which do not significantly over-express disease-associated proteins, have more subtle phenotypes that are challenging to work within the context of drug screening. Multiple phenotypic readouts across different *in vitro* and *in vivo* model systems that replicate different aspects of the disease phenotype could help to overcome this. Compounds which show reproducibility across different model systems and phenotypes will help to prioritize the best candidates to take forward for further clinical development.

### 4.2. Blood brain barrier permeability

Identifying compounds with the ability to cross the blood–brain barrier (BBB) is assumed to be vital when considering targeting pathology that is primarily CNS-based, which includes ALS. The BBB separates the brain and spinal cord from blood components and the peripheral immune system. The integrity of the BBB is essential for protection from blood-borne pathogens and toxins and the maintenance of the brain microenvironment, which is crucial in allowing neuronal networks to function optimally [151]. However, the BBB has also created significant challenges in developing CNS-penetrant therapies. Poor penetration efficacy across the BBB is one of the main factors underlying the high failure rate of drugs designed to target CNS disorders [60]. In drug repurposing the medicinal chemistry properties of many drugs have already been investigated to some extent, making it easier to identify drugs that are already known to penetrate the CNS. However, it is important to validate the ability of drugs to cross the BBB and reach the target site of action prior to clinical testing. This could be achieved through the use of *in vitro* BBB models and using *in vivo* animal models [152].

There has been progress in using targeted ultrasound to temporarily disrupt the BBB and allow inherently restricted small molecules or biomolecules into the brain has great promise in opening up the possibilities for repurposing [153]. The use of focused ultrasound and microbubbles to increase the permeability of an anti-TDP43 monoclonal antibody across an ALS iPSC-derived endothelial model of the BBB highlights how this technique can be translated from the preclinical setting [154]. A focused ultrasound device to deliver the peripherally restricted anti-cancer agent carboplatin to the CNS of recurrent glioblastoma patients is in trial [155]. If successful in cancer treatment, temporary disruption of the BBB with ultrasound will have clear potential for repurposing treatments for ALS.

### 4.3. Combinatorial treatments

It is widely accepted that a single drug may be insufficient to induce clinically significant alterations in ALS disease progression (with the exception of precision medicine approaches such as genetic therapies targeting *FUS* and *SOD1*). Given this, there is a need to develop robust approaches to combinatorial screening and validation preclinically and the methodology for combinatorial drug trials. The ideal combination of drugs would target different aspects of the pathophysiological cascade, underlining the need to develop a better understanding of the order of events in ALS. However, which pathways are primary drivers of ALS pathogenesis, and therefore are most important to target therapeutically, remains unclear. This lack of clarification may somewhat underlie the failure of the combined treatment, sodium phenylbutyrate and taurursodiol, which targets endoplasmic reticulum stress and mitochondrial dysfunction, respectively. Whilst the combined treatment was found to reduce neuronal death and ameliorate pathological features in experimental models of ALS and other neurodegenerative diseases [156–163], treatment was found to have no significant effect on slowing disease progression in a Phase III study [86].

The fact that some drugs may have little effect when given alone does not exclude the possibility they may act in synergy to achieve a meaningful therapeutic effect when given in combination with other drugs. The scale of research required to systematically investigate various combinations of therapies, both in preclinical models and following on in clinical trials would require large international coordination. It will be useful to learn from combinatorial analysis of drugs in the context of other disease areas such as cancer, by adopting an adaptive study design to enable simultaneous testing of multiple treatment regimens. This approach has been used in the I-SPY 2 (investigation of serial studies to predict your therapeutic response with imaging and molecular analysis) TRIAL for breast cancer therapeutics [164]. Individual treatment arms can be halted or expanded based on interim results (such as changes in biomarkers) allowing trials to be adapted as they progress. The heterogeneous nature of neurodegenerative diseases such as ALS favors such adaptive trials, which can be subjected to Bayesian methods of adaptive randomization to allocate drugs to different subsets of patients based on clinical, genetic, pathological, phenotypic and biomarker data [165].

### 4.4. Human surrogate markers from experimental medicine

Undoubtedly a major barrier to therapy development in ALS has been the lack of a reliable, sensitive *human* biomarker of disease activity, ideally predictable in terms of its natural history prior to any intervention, and at a more individualized or small group level at least.

NfL is a structural component of the axonal cytoskeleton, released into the cerebrospinal fluid (CSF) in response to neuronal damage from a range of causes [166]. It can now be reliably detected more conveniently in the blood with strong CSF-level correlation [167]. NfL is a nonspecific biomarker of axonal damage, but its levels are generally proportional to the ‘aggressiveness’ of the disease process [168]. Unsurprisingly, ALS is associated with significantly elevated levels of NfL, reflecting its catastrophic median survival. However, lower levels are seen in those with more slowly progressive disease, overlapping with other neurological disorders. While this greatly limits the independent *diagnostic* value of NfL [169], there is a stronger correlation of NfL level with the rate of disability progression as measured using the revised ALS Functional Rating Score (ALSFRS-R), a change in the downward slope of which is the current gold standard, albeit insensitive, outcome measure in ALS trials. Furthermore, within individual patients, NfL levels remain relatively stable over time [167,170]. This has brought NfL to the forefront as a *prognostic* and *pharmacodynamic* biomarker in ALS [170].

In a further key development, proof of principle that a fall in NfL levels across a treatment group is *predictive* of later clinical benefit has come from trials of the ASO therapy, Tofersen. This targets a reduction in CSF SOD1 protein for the ~2% of ALS associated with pathological variants in *SOD1*. In a Phase III clinical trial in 108 patients, treatment led to rapidly reduced SOD1 protein levels, accompanied by ~50% reduction in mean NfL level. While no clear *clinical* benefit (significant difference in the ALSFRS-R slope between groups) was observed after 6 months, the slopes continued to separate over longer period of follow-up [38], with data from a subsequent open-label extension phase leading to FDA approval of Tofersen, in which NfL was uniquely acknowledged to have reached the very high bar of a ‘reasonably likely surrogate marker of clinical benefit.’

Experimental Medicine is a term used to refer to human-based studies that may establish proof of concept in terms of a particular biochemical pathway or build confidence in the potential for a drug through establishing ‘signals’ of likely clinical benefit. This latter role can then significantly ‘de-risk’ the large financial and time investment inherent to a placebo-controlled trial. In ALS, it requires approximately 300 patients over at least 9 months to demonstrate clinical benefit using the slope of ALSFRS-R decline. While NfL levels are increasingly being incorporated as secondary outcome measures into Phase II and III studies in ALS, there is growing interest in exploiting it for more rapid drug ‘screening,’ where a pre-specified fall in mean NfL levels can define a ‘go’ or ‘no-go’ decision for definitive trial. The EXPErimental medicine Route To Success (EXPERTS-ALS) is a UK National Institute of Health & Care Research-funded proposal for a Bayesian NfL-driven open-label platform trial in ALS comparing up to three repurposed drug arms at any one time. It is powered to detect a 30% or greater reduction in mean NfL (‘go’) or to declare futility (‘no-go’) within 3–6 months using 30–75 ALS patients each taking drug for a maximum of 6 months (www.experts-als.uk). This approach cannot prove clinical benefit but any drugs showing significant NfL reduction can be prioritized for definitive Phase III placebo-controlled study with the prior knowledge of an established human surrogate marker of likely clinical benefit.

There are important caveats to this approach, which will need to be explored through experience. While the chance of missing an important therapeutic benefit that is somehow not associated with a lowering of NfL levels is considered very low (intuitively based on data from many large ALS clinic-based cohorts linking NfL levels to prognosis), there is the potential for a transient-continued rise in NfL levels during the early symptomatic period before stability is reached, and the potential for drugs to reduce NfL clearance (and so lead to higher levels) through unexpected pathways which might not necessarily reflect disease worsening [171].

## 5. Conclusions

The search for new effective treatments for ALS is on-going. Although not without its challenges, drug repurposing offers an alternative approach to drug discovery that overcomes some of the limitations of *de novo* drug discovery. Advances in disease modeling and technological developments have created new opportunities to interrogate disease–drug interactions and identify repurposed drugs that show potential as new ALS therapeutics. However, a collaborative effort across academia, industry and wider stakeholders will be required to meet the inevitable challenges known to hamper drug repurposing.

## 6. Expert opinion

Drug repurposing is not a new concept, but it is one that has gained significant momentum. Renewed interest stems in part from challenges posed by traditional drug discovery approaches, including lengthy drug discovery pipelines, high risks of failure and soaring costs. However, there is also increased recognition, in the context of a growing list of molecular pathway derangements in ALS, of potential for hidden ‘off target’ benefits, worthy of exploration and with novel high-throughput screening tools. Technological innovations over the last decade have yielded new drug repurposing tools that can be used to scrutinize drug–disease interactions at a previously unprecedented scale. The combined use of *in silico* methods, AI, omics technologies and high-throughput screening provides the opportunity to examine drug repurposing from multiple angles and deliver a substantial body of evidence to help guide the selection of candidates for further investigation.

**Figure 2.**
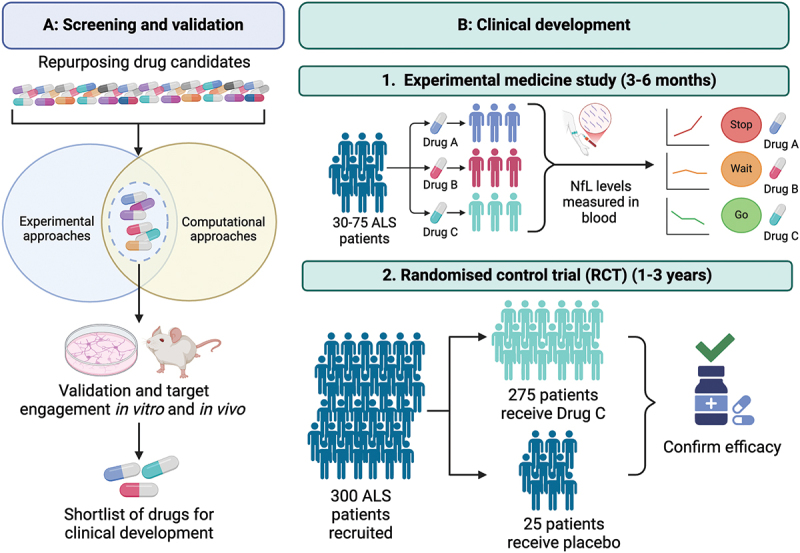
A proposed framework for the screening, validation and clinical development of repurposed drugs in ALS. (a) Screening of drug candidates for repurposing using experimental and computational approaches to identify drugs consistently identified as ‘hits’. Validation of these drugs in vitro and in vivo to confirm target engagement and generate a shortlist of drugs for further development. (b) Initial clinical investigation in short experimental medicine studies utilising human biomarkers (neurofilament light (NfL)), to identify the most promising candidates to enter longer-term, large-scale, randomised, placebo-controlled clinical trials.

ALS is well suited to drug repurposing for many reasons, including the multifactorial nature of disease pathogenesis. However, stringent preclinical research will be required, as these experiments lay the foundations for future research and ultimately determine which candidates are brought forward for further development. Early screening approaches should draw upon a range of experimental and computational techniques to select the most promising drugs. Given the advantages and limitations of these different techniques, drugs that are consistently identified as positive ‘hits’ will provide the strongest indicator of their repurposing potential in ALS (Figure 2). Subsequently, the selection and validation of these drugs should be performed across a range of *in vivo* mouse models, reflecting the different genetic and pathophysiological aspects of the disease. Over-reliance on the SOD1^G93A^ transgenic over-expression mouse model of ALS has delivered minimal success. Advances in genetic engineering have allowed the development of more complex, physiologically more relevant mouse models (including knock-ins and bespoke complex models) that could be used to reflect broader subtypes of ALS [172]. Using such models will be crucial to demonstrate target engagement and avoid missed opportunities, as it may be that while one drug shows promise in a particular subtype of the disease, it may be less beneficial for others.

Secondly, BBB permeability is an important consideration, even in the early preclinical stages. Although not impossible, it can be extremely challenging to modify such properties, and attempting to do so could be risky and create further delays in the drug development process [173]. Furthermore, combinatorial treatments are likely to feature heavily in the future of ALS drug discovery, providing the potential to target multiple pathophysiological mechanisms simultaneously. Whilst drug discovery pipelines for the analysis of combinatorial treatments pose a greater logistical challenge, drawing upon expertise from different diseases (such as cancer) where such approaches have been successfully implemented will be immensely valuable.

Clinical heterogeneity in ALS poses a significant challenge to drug repurposing. Those with ALS present with a range of clinical upper and lower motor neuron system involvement, varying rates of disease progression and diverse genetic and molecular hallmarks [2]. In anticipation of a more Personalized Medicine approach to therapy, *post hoc* analysis has been used to isolate the effects of treatments in a sub-set of ALS patients. Stratification based on genetic status yielded unexpected results in the case of a therapeutic trial of lithium. Despite an overall negative result, the lithium arm was found to have beneficial effects in a sub-set of patients with *UNC13A* variants [174]. A Phase III trial for lithium carbonate is now recruiting patients specifically with *UNC13A* variants to further investigate the efficacy and safety of lithium in this patient group (NCT06008249) (Table 1). Monitoring individual differences in response to treatment will require reliable biomarkers to track subtle biological changes in disease state which may indicate subsequent clinical change. There will be greater reliance on the use of human surrogate markers to guide ‘go’ and ‘no-go’ decisions in clinical trials. Nfl is currently the most reliable candidate, but future research could identify more specific markers of disease activity that could be used alongside Nfl to track response to treatments.

Drug repurposing still faces many logistical barriers, often underestimated by researchers. There are also several regulatory, legal and intellectual property hurdles that need consideration [175,176]. Furthermore, drug repurposing, whilst more economically viable than traditional drug discovery approaches, still requires significant investment. Investment comes from a variety of sources including academia, pharmaceutical companies, biotechnology companies and research charities. Whilst united in the common aim of identifying promising drugs for repurposing, these different institutes have different capabilities, fields of expertise and may have competing interests. Fostering collaboration between these different institutes through shared funding schemes, disclosing details of off-target effects and sharing drugs, will be essential to combine the strengths of each party and optimize the potential of drug repurposing. Academia provides a source of innovation, where basic research leads to the identification and validation of novel therapeutic targets [177]. Industry partners provide significant expertise in drug development processes, particularly in the areas of drug metabolism, pharmacokinetics and pharmacodynamics, drug manufacturing, formulation and delivery, and regulatory affairs [178]. Collaborations between industry and academia have proven successful in the past, for example with the kinase inhibitor, alpelisib. Originally indicated for the treatment of breast cancer associated with PIK3CA mutations, the compound was shared with academic researchers to investigate the effects of alpelisib in patients with PIK3CA-related overgrowth spectrum (PROS). After demonstrating promising results in PROS mouse models and subsequently in 19 PROS patients [179], alpelisib was granted accelerated approval in 2022 for the treatment of severe manifestations of PROS [180]. This example highlights how collaborations between academia and industry can be successfully harnessed to accelerate drug repurposing. There are several examples of initiatives aimed at increasing collaboration between academia and pharmaceutical companies to investigate drug repurposing opportunities. These include the partnership between the UK’s Medical Research Council (MRC) and the US NIH-National Centre for Advancing Translational Sciences (NIH-NCATS) in collaboration with several pharmaceutical companies [181]. Furthermore, the UK’s LifeArc has launched a £5 million pound drug repurposing program for ALS to foster collaborations with researchers in academia.

There are many challenges remaining, however, if executed appropriately, drug repurposing offers an important route for ALS drug discovery and the development of effective therapeutics.

## Article highlights

- Drug discovery in ALS has been particularly challenging due to the complex pathophysiology of the disease and the lack of preclinical models that provide clear predictive therapeutic value.
- Drug repurposing offers a complementary approach to expensive and time-consuming traditional drug discovery approaches, by reevaluating the potential of existing drugs for the treatment of new disease indications.
- Drug repurposing utilizes a broad range of techniques spanning experimental and computational approaches to interrogate drug–disease interactions.
- Several repurposed drugs are currently in clinical trials for ALS, although to date no repurposed drugs have been successfully translated into new treatments for patients in the clinic.
- Successful drug repurposing in ALS will rely on stringent preclinical research to select the most promising candidates for further development coupled with innovative experimental medicine studies to help bridge the translational gap between preclinical and clinical studies.
- Fostering collaboration between academia, industry and wider stakeholders will be key to accelerating drug repurposing in ALS by harnessing the strengths of each party.

## Abbreviations

ABPP: Activity-based protein profiling
AD: Alzheimer’s disease
ALS: Amyotrophic lateral sclerosis
ALSFRS: ALS functional rating score
ALSFRS-R: ALS functional rating score revised
AI: Artificial intelligence
ASO: Antisense oligonucleotide
BBB: Blood brain barrier
CCCP: Compound-centric chemical proteomics
CETSA: Cellular thermal shift assay
CMap: Connectivity map
CNS: Central nervous system
CSF: Cerebrospinal fluid
EHR: Electronic health records
qQTLs: Expression quantitative trait loci
ER: Endoplasmic reticulum
FDA: U.S Food and Drug Administration
FTD: Frontotemporal dementia
FUS: Fused in sarcoma
GWAS: Genome wide association study
iPSC: Induced pluripotent stem cell
iPSC-MN: Induced pluripotent stem cell-derived motor neuron
LINCS: Library of integrated network-based cellular signatures
MND: Motor neuron disease
MR: Mendelian randomization
NfL: Neurfilament light
PGK1: Phosphoglycerate kinase 1
SOD1: Superoxide dismutase 1
SNP: Single nucleotide polymrphisms
TARDBP: TAR-DNA binding protein 43
TNF: Tumour necrosis factor
TWAS: Transcriptome wide association study
